# Productive Performance and Egg Quality Traits of Exotic Chicken Breeds Reared Under Traditional Management System in Misrak Silte District, Central Ethiopia

**DOI:** 10.1155/sci5/1200900

**Published:** 2026-02-23

**Authors:** Mohammed Sirmolo, Aleme Asresie, Sefa Salo, Yilkal Tadele

**Affiliations:** ^1^ Misrak Silte District Agriculture and Rural Development Office, Silte Zone, Ethiopia; ^2^ Department of Animal Sciences, Wollo University, Dessie, Ethiopia, wu.edu.et; ^3^ Department of Animal Sciences, Wachemo University, Hossana, Ethiopia, wcu.edu.et; ^4^ Department of Animal Sciences, Arba Minch University, Arba Minch, Ethiopia, amu.edu.et

**Keywords:** constraints, egg production, husbandry, smallholder farmers

## Abstract

This study assessed feeding practices, egg production performance, egg quality, and production constraints of exotic chicken breeds (Sasso and Bovans Brown) reared under traditional management systems in Misrak Silte District, Central Ethiopia. Purposive and random sampling was used to select study kebeles and 316 households. Data were collected on household characteristics, husbandry practices, egg production performance, and egg quality traits, and 160 eggs were sampled for quality evaluation. Results showed that 87.7% of respondents provided supplementary feed, mainly household food leftovers (70.8%) and grain leftovers (17.5%). Most respondents (60.76%) housed chickens only at night. The average age at first lay (days) for Sasso chickens in the midland and lowland was 170.35 ± 14.54 and 149.06 ± 8.21, respectively, while Bovans Brown recorded 180.69 ± 12.17 and 168.22 ± 8.73. Annual egg production (eggs/hen/year) was higher in the midland than the lowland for both breeds (*p* < 0.05), with Sasso producing 167.9 ± 8.48 and 149.6 ± 6.92 eggs and Bovans Brown producing 145.2 ± 4.04 and 136.6 ± 4.73 eggs, respectively. Agroecology significantly affected (*p* < 0.05) both external and internal egg quality traits, and higher values for most quality parameters were recorded in the midland agroecology than in the lowland. Disease, feed shortage, predator attacks, and lack of capital were the major constraints in both agroecologies. In conclusion, productive performance and egg quality traits of exotic chickens under traditional management were significantly influenced by breed and agroecology, with Sasso chickens and the midland agroecology showing superior performance.

## 1. Introduction

Ethiopia, with a diverse agroecological zones and favorable environmental conditions, owned about 57 million heads of chicken which comprises 88.19% indigenous breeds, 6.45% hybrids, and 5.36% exotic breeds [1]. However, the productivity of Ethiopian indigenous chickens is low [2].

Over the years, in order to improve the overall productivity, different breeds of exotic chickens were distributed to the smallholder farmers across the country including Southern Ethiopia. However, lack of recorded data on husbandry practices and productivity potential of different exotic breeds across agroecological variations (comparative studies) makes it difficult to assess the importance and contributions of the past attempts to improve the sector [2, 3].

Chicken production is widely being promoted in rural, peri‐urban, and urban areas of the southern region as a means of job opportunity and poverty reduction [4]. Since 2014, Sasso and Bovans Brown (BB) chickens have been distributed through government extension service in rural areas of the country [5].

It has been reported that Misrak Silti District has the potential of poultry production from exotic chicken, and chicken production is an important activity for sustaining the livelihood of farming community. Therefore, understanding the existing production practices, productivity, and constraints is important to devise improvement interventions. However, there have been limited studies undertaken to assess chicken husbandry practices, egg production performance, quality, and associated constraints. Farmers’ husbandry practices, egg production abilities, and egg quality of Sasso and BB chickens in the district remain undocumented. Therefore, this research was initiated with the objective to assess husbandry practices, egg production, and quality and associated constraints under village‐based production systems in different agroecologies of the district.

## 2. Materials and Methods

### 2.1. Description of the Study Area

The study was conducted in Misrak Silti District, located in the Silte Zone of Central Ethiopia, at approximately 7°49′55.88″ N latitude and 38°16′7.39″ *E* longitude. The district lies at an altitude ranging from 1833 to 2200 m above sea level. The area experiences a mean annual temperature of 14°C–23°C and receives annual rainfall ranging from 875 to 1213 mm.

Misrak Silti District is characterized by two agroecological zones: mid‐altitude and low‐altitude, covering 83.33% and 16.67% of the area, respectively. Administratively, the district comprises 12 kebeles, of which 10 are mid‐altitude and 2 are low‐altitude. The district covers a total area of 11,098.27 ha and has an estimated human population of 171,950. Crop production, dominated by maize, barley, wheat, and pepper, along with livestock rearing, constitutes the main livelihood of the rural population [6].

### 2.2. Sampling Technique and Sample Size Determination

Misrak Silti District was selected purposively from the Silte Zone based on chicken production potential and distribution of exotic chicken breeds [6]. The district was stratified into midland and lowland agroecologies, which comprise 10 and 2 kebeles, respectively. From these, five kebeles (50%) were randomly selected from the midland agroecology, while both kebeles (100%) were included from the lowland agroecology to ensure adequate representation of this smaller agroecological zone. Selection of kebeles was based on the presence of exotic chicken populations, production potential, and accessibility to all‐weather roads. Individual households having at least 5 exotic chickens in the selected kebeles were identified and listed. Then, respondent households were randomly selected from the list.

Sample size for survey study was computed based on the Yemane [7] formula at 95% confidence level and 5% level of precision. *n* = N/1 + N (e)^2^ = 1505/1 + 1505 (0.05)^2^ = 316.01–316, where “*n*” is the sample size, “*N*” is the total population/total households having exotic chickens, and “e” is the level of precision. The total number of respondents for this study was 316 (222 from midland and 94 from lowland).

### 2.3. Data Collection Methods

A semistructured questionnaire was used to collect survey data. Primary data on household characteristics (sex, family size, and level of education), housing, feeds and feeding, number of egg produced/hen/year, and constraints were collected.

### 2.4. Egg Quality Evaluation

A total of 160 fresh eggs were collected from adult laying hens for evaluating egg quality parameters. Forty (40) eggs per breed were collected from each agroecology, making a total of 80 eggs for both Sasso and BB breeds. Due to financial constraints (egg purchasing) and transportation requirements, we limit number of eggs for quality analysis. Eggs were collected, during the dry season, from hens of similar age groups for evaluation of external and internal egg quality traits. The ages of the hens were estimated to be approximately 8 months, based on information provided by the respondents. During collection, we have tried to maintain uniformity by avoiding extremely small and large eggs. Eggs were collected on the same day they were laid. The collected eggs were then transported to the Debre Zeit Agricultural Research Center for quality analysis.

### 2.5. Evaluation of External and Internal Egg Quality Traits

External egg quality traits such as egg weight, length, width, shape index, shell weight, shell thickness, and shell ratio were determined. Egg weight was measured individually using a digital sensitive balance (Model: Sartorius Entris 323‐1S, Sartorius AG, Germany) with a sensitivity of 0.01 g [8].

To calculate the shape index, the length (mm) and breadth (mm) of each egg were measured using a digital caliper meter. The formula: egg shape index (%) = egg length (mm)/egg width (mm) × 100, was used [9].

Eggs were broken onto a glass‐covered table; albumen and yolk were separated and weighed. Albumen and yolk heights were measured at its widest part using a tripod micrometer (dial compressor gauge). Yolk diameter was measured horizontally using a digital caliper meter. Yolk color was determined by comparing it with the Roche Yolk Color (RYC) Fan, which contains 15 scales with a standard colorimetric system [10]. The cleaned egg shell weight was weighed using a digital balance. The egg shell thickness was measured using a digital caliper meter determined by taking the average thickness of the large end, center, and narrow ends.

The yolk index was measured by dividing the height of the yolk by its diameter [11]. Haugh unit (HU) was calculated as the ratio between egg weight and albumen height (mm). The individual HU was calculated using the formula developed by Haugh [12]. HU = 100 Log (AH–1.7 EW^0.37^ + 7.6), where HU = Haugh unit, AH = albumen height in millimeters, and EW = egg weight in grams.

### 2.6. Data Management and Statistical Analysis

The survey data were analyzed using the Statistical Package for Social Sciences, Version 25 [13]. Data on external and internal egg quality traits parameters were analyzed by two‐way ANOVA using the SPSS. Whenever ANOVA showed statistically significant difference among means, the mean values of egg samples were compared by using the least significant difference (LSD) test at 5% significance level. The model used to compare egg quality traits at different agroecology zones was as follows.

Yijk = *μ* + Ai + Bj + Ai × Bj + eijk, where Yijk = the observed *k* ^th^ variable in the *i* ^*t* *h*^ agroecology and *j* ^th^ breed, *μ* = overall mean of the observed variables, Ai = effect due to *i* ^*t* *h*^ agroecology (*i* = lowland, midland), Bj = effect due to *j* ^*t* *h*^ breed of chickens (*j* = Sasso and BB), Ai ∗ Bj = effect due to interaction between *i* ^*t* *h*^ agroecology and *j* ^*t* *h*^ breed, and eijk = random residual error.

## 3. Results

### 3.1. Household Characteristics

The household characteristics of chicken farmers are presented in Table 1. The majority (65.2%) of poultry farmers were females. This indicated that females are actively participating in exotic chicken production than males in the study areas. Thismay be attributed to the fact that chickens are typically kept close to the home, making it easier for women to combine poultry care with childcare and other domestic tasks. Regarding educational levels, higher proportions (60.8%) of the respondents are illiterate.

**TABLE 1:** Household characteristics of respondents in the study areas.

Household characteristics		Agroecology		Overall (*n* = 316)
		Midland (*n* = 222)	Lowland (*n* = 94)	
Sex (%)	Male	34.70	35.10	34.80
	Female	65.30	64.90	65.20

Educational status (%)	Illiterate	56.30	71.30	60.80
	Elementary school	39.20	28.70	36.10
	High school	3.60	0.00	2.50
	College and above	0.90	0.00	0.60

Family size (%)	2–4	24.30	28.70	25.6
	5–7	64.90	58.80	63.00
	8–10	10.80	12.80	11.40

*Note: n* = number of respondents.

Education helps to better understand and adopt improved poultry management practices such as feeding, disease control, and vaccination schedules which directly improve the productivity of chickens. Most (63%) of the respondent households have 5–7 numbers of persons in their family.

### 3.2. Feeding and Feeding Practices

The feeds and feeding practice of chickens in the study district are presented in Table 2. The feeding practices of chicken in the study areas revealed that most of the respondents (87.7%) practice scavenging feeding with additional supplements. The majority of the respondents (78.4%) in the study area provide supplement feed to their chicken once a day. Exotic chickens were managed under traditional systems similar to local breeds; however, their higher production potential may make them more sensitive to feed quality and supplementation.

**TABLE 2:** Feeding practices of exotic chicken breeds in the study areas.

Variables		Agroecology		Overall (*n* = 316)
		Midland (*n* = 222)	Lowland (*n* = 94)	
Feeding practices (%)	Only scavenging	8.60	29.80	19.20
	Scavenging with additional supplement	91.40	70.20	80.80

Supplementation time (%)	Morning only	72.00	95.00	83.50
	Afternoon only	10.00	5.00	7.50
	Morning and afternoon	18.00	0.00	9.00

*Note: n* = number of respondents.

### 3.3. Feed Supplements

The main supplementary feeds provided to exotic chickens in the study area are shown in Table 3. Food leftovers were ranked first in both midland and lowland agroecologies. Other important supplements included wheat grain, maize and sorghum, and grain leftovers (weed seeds, husks, and insect‐damaged grains). The overall ranking of supplements was as follows: food leftover > wheat grain > maize and sorghum > grain leftover.

**TABLE 3:** Feed supplements in the study area as ranked by respondents.

Feed supplements	Agroecology				Overall (*n* = 316)	
	Midland (*n* = 222)		Lowland (*n* = 94)			
	Index	Rank	Rank	Rank	Index	Rank
Wheat grain	0.27	2^nd^	0.22	3^rd^	0.24	2^nd^
Food leftover	0.38	1^st^	0.41	1^st^	0.395	1^st^
Grain leftover	0.25	3^rd^	0.08	4^th^	0.17	4^th^
Maize and sorghum	0.1	4^th^	0.29	2^nd^	0.195	3^rd^

*Note: n* = number of respondents.

### 3.4. Housing Practices

Housing is an important husbandry practice in chicken production. Chicken housing practices in the study area are presented in Table 4. About 60% of the respondents use only night‐time shelter for their chicken. Respondents (25.95%) house their chicken with the family, and only few of them construct separate chicken houses. All age groups and breeds of chicken are housed together.

**TABLE 4:** Chicken housing practices.

Housing types for chickens (%)	Agroecology		Overall (*n* = 316)
	Midland (*n* = 222)	Lowland (*n* = 94)	
Share family house	23.80	31.00	25.95
Separate chicken house	14.50	11.00	13.29
Night‐time shelter/under the basket	61.70	58.00	60.76

*Note: n* = number of respondents.

### 3.5. Productive Performance of Exotic Chicken Breed

Productive performances of exotic chicken breeds are presented in Table 5. In the midland agroecology, both Sasso and BB breeds exhibited a significantly (*p* < 0.05) longer age at first egg (AFE) compared to their counterparts in the lowland agroecology. This variation might be due to the feed availability and temperature differences. In the higher altitudes (midland), considerable portion of feed energy may be diverted to maintain body temperature rather than growth and reproductive development.

**TABLE 5:** Performance of exotic chicken breeds in the study areas (mean ± SD).

Variables	Agroecology		*p* value
	Midland	Lowland	
*Performance of Sasso breed*			
Age at first egg in days	170.35^a^ ± 14.54	149.06^b^ ± 8.21	< 0.001
Total number of eggs laid per hen/year	167.9^a^ ± 8.48	149.6^b^ ± 6.92	< 0.001
Adult female body weight (kg)	2.68 ± 0.311	2.60 ± 0.283	0.056

*Performance of Bovans Brown breed*			
Age at first egg in days	180.69^a^±12.17	168.22^b^ ± 8.73	< 0.01
Total number of eggs per hen/year	145.2^a^±4.04	136.6^b^ ± 4.73	< 0.001
Adult female body weight (kg)	2.48 ± 0.22	2.41 ± 0.24	0.298

Abbreviation: kg = kilograms.

^a,b^Means with different superscripts in a row are significantly different at *p* < 0.05.

### 3.6. External Egg Quality Traits of Exotic Chicken

Evaluated external egg quality traits include egg weight, egg length, egg width, shape index, shell thickness, shell weight, and egg shell ratio (Table 6). External egg quality traits were significantly (*p* < 0.05) different between breeds but not affected by agroecology (*p* > 0.05). Significantly (*p* < 0.05) higher egg width, egg shell index, egg shell weight, and egg shell ratios were recorded for BB than Sasso T44 breed.

**TABLE 6:** External egg quality traits of exotic chicken in the study area (mean ± SE).

Variables	External egg quality traits						
	EW (g)	EL (mm)	EWD (mm)	ESI (%)	ESW (g)	EST (mm)	ESR (%)
*Agroecology*							
Midland	59.41 ± 0.54	55.28 ± 0.27	42.40 ± 0.16	76.96 ± 0.31	7.27 ± 0.11	0.41 ± 0.004	12.27 ± 0.18
Lowland	58.38 ± 0.76	54.36 ± 0.39	41.98 ± 0.23	77.32 ± 0.43	6.86 ± 0.15	0.42 ± 0.06	11.77 ± 0.26

*Breeds*							
Sasso breed	58.37 ± 0.66	55.08 ± 0.34	41.77^b^ ± 0.2	76.05^b^ ± 0.37	6.59^b^ ± 0.13	0.40 ± 0.05	11.30^b^ ± 0.22
Bovans Brown	59.42 ± 0.66	54.55 ± 0.34	42.61^a^ ± 0.2	78.22^a^ ± 0.37	7.54^a^ ± 0.13	0.42 ± 0.05	12.74^a^ ± 0.22

*p* *values*							
Agroecology (A)	0.274	0.06	0.15	0.49	0.03	0.09	0.11
Breed (B)	0.264	0.27	0.004	< 0.001	< 0.001	0.07	< 0.001
A ∗ B	0.149	< 0.001	< 0.001	< 0.001	0.79	0.21	0.63

Abbreviations: EL = egg length, ESI = egg shape index, ESR = egg shell ratio, EST = egg shell thickness, ESW = egg shell weight, EWD = egg width, and EW = egg weight.

^a,b^Means with different superscripts in a row are significantly different at *p* < 0.05.

### 3.7. Internal Egg Quality Traits of Exotic Chicken

Evaluation of internal egg quality traits includes albumen height, albumen weight, yolk height, yolk weight, yolk width, yolk color, yolk index, and HU (Table 7). The effect of agroecology was significant (*p* < 0.05) for all internal egg quality traits except yolk index and yolk color. Similarly, the effect of breed was significant (*p* < 0.05) for all internal egg quality traits except for yolk height, yolk diameter, and yolk index.

**TABLE 7:** Internal egg quality traits of exotic chicken in the study area (mean ± SE).

Variables	Internal egg quality traits							
	YH	YD	YI	AH	YW	YC	AW	HU
*Agroecology*								
Midland	17.25 ± 0.13	39.88 ± 0.17	43.27 ± 0.35	7.35 ± 0.07	14.82 ± 0.19	9.65 ± 0.22	40.27 ± 0.52	85.94 ± 0.47
Lowland	16.50 ± 0.18	39.28 ± 0.24	42.08 ± 0.49	6.50 ± 0.10	16.90 ± 0.27	10.00 ± 0.31	34.72 ± 0.73	80.52 ± 0.66

*Breed*								
Sasso	17.10 ± 0.16	39.79 ± 0.21	43.06 ± 0.43	6.58 ± 0.09	17.36 ± 0.23	10.45 ± 0.27	34.64 ± 0.63	81.09 ± 0.57
Bovans Brown	16.65 ± 0.16	39.37 ± 0.21	42.29 ± 0.43	7.27 ± 0.09	14.36 ± 0.23	9.20 ± 0.27	40.36 ± 0.63	85.36 ± 0.57

*p* *values*								
Agroecology (A)	0.001	0.04	0.05	< 0.001	< 0.001	0.36	< 0.001	< 0.001
Breed (B)	0.05	0.15	0.21	< 0.001	< 0.001	0.001	< 0.001	< 0.001
A ∗ B	0.51	0.002	0.02	< 0.001	< 0.001	0.24	< 0.001	< 0.001

*Note:* YH = yolk height (mm), YD = yolk diameter (mm), YI = yolk index (%), AH = albumin height (mm), YW = yolk weight (g), and AW = albumin weight (g).

Abbreviations: HU  =  Haugh unit; YC  =  yolk color.

### 3.8. Constraints of Exotic Chicken Poultry Production

The major constraints of exotic chicken production in the study areas are presented in Table 8. The major constraints of chicken in the study areas were presence of disease (1^st^), shortage of feed (2^nd^), predator attack (3^rd^), and lack of capital (4^th^).

**TABLE 8:** Constraints of exotic chicken poultry production in the study area.

Constraints	Agroecology					
	Midland		Lowland		Overall	
	Index	Rank	Index	Rank	Index	Rank
Feed shortage	0.295	2^nd^	0.31	1^st^	0.3025	2^nd^
Presence of disease	0.371	1^st^	0.28	2^nd^	0.3255	1^st^
Predator attack	0.25	3^rd^	0.23	3^rd^	0.24	3^rd^
Lack of capital	0.084	4^th^	0.18	5^th^	0.132	5^th^

## 4. Discussion

The higher proportion of female respondents in this study aligns with Adem and Teshome [14], who reported that women play a major role in chicken production in Yeki District, Southwestern Ethiopia. Similar findings were reported by Habtamu et al. [3] in the Amhara Region and Tsadik et al. [15] in Central Oromia. Conversely, Fasil et al. [16] noted a higher proportion of males (59.3%) engaged in chicken rearing in Jama District, South Wollo Zone, suggesting that gender roles in poultry production may vary across regions and production systems.

About 60.8% of respondents were illiterate, consistent with studies in Keffa, Abobo, and Gambela Zuria districts [17]. Limited education may reduce adoption of improved husbandry practices, whereas educated farmers are more likely to implement technologies that enhance productivity. Larger families may provide more labor for chicken care and greater access to household food leftovers, while also motivating households to rear more chickens for home consumption.

Most farmers supplemented scavenging with additional feed, as reported by Tadesse et al. [18] and Salo et al. [19]. The main supplementary feeds were food leftovers, grain residues, maize, sorghum, and wheat, consistent with observations in other parts of Ethiopia [3, 20–22].

Housing practices were rudimentary, with 60.76% of respondents providing shelter only at night, often within the family house (65%), and releasing chickens for scavenging during the day [23]. Low construction of separate chicken houses has also been reported in other regions [15, 24]. Such practices may protect chickens from predators but can increase disease risk.

The AFE for Sasso chickens in this study was comparable to AFE reported in Hawassa and Wolaita [25], indicating that environmental and management factors influence sexual maturity. Egg production per hen per year was significantly higher in midland than lowland agroecologies (*p* < 0.001), likely due to favorable temperatures that reduce heat stress and improve feed intake. The overall egg production and weight for Sasso and BB chickens were within ranges reported by Serkalem et al. [26], Birtukan et al. [27], and Desalew et al. [4].

External egg quality traits were influenced by agroecology, reflecting differences in feed quality and availability [28]. Genetic factors affected egg weight, shape index, and shell ratio, while shell thickness depended on calcium deposition. Internal egg quality traits such as albumin height, yolk height, and color were influenced by both genotype and feed quality [29–31]. Body weight was significantly correlated with egg weight, supporting previous findings [32].

Regarding production constraints, diseases were the major challenge, in agreement with reports from Wolaita, North Gondar, and Hadiya zones [19]. Feed shortages and lack of improved breeds were also reported as key constraints in Western Amhara [29].

Overall, the findings demonstrate that gender, education, family size, feed management, housing, agroecology, and breed significantly influence chicken productivity. Addressing these factors through improved management, feed provision, and disease control could enhance the performance of exotic chicken breeds under smallholder conditions.

## 5. Conclusion

Exotic chicken production in Misrak Silti District is mainly practiced by female farmers, with most providing supplementary feeds such as food leftovers, grain leftovers, maize, sorghum, and wheat. Productivity and egg quality were influenced by breed and agroecology, with Sasso chickens and midland agroecology performing better. Major constraints included disease, feed shortages, predator attacks, and lack of capital. Training on improved housing, feeding, and disease control, along with interventions to alleviate feed shortages and improve access to credit, is recommended. A limitation of the study is the lack of data on flock structure and the purpose of keeping chickens [33, 34].

## Author Contributions

All authors contributed equally to this research manuscript.

## Funding

No external funds were received for this study.

## Ethics Statement

All the respondent households were informed about the objectives of the study. They verbally agreed to participate as part of informed consent.

## Conflicts of Interest

The authors declare no conflicts of interest.

## Data Availability

Data are available from the authors upon reasonable request.
